# Exploring the mechanism of Liang Xue Wu Hua Tang in the treatment of rosacea via network pharmacology and molecular docking

**DOI:** 10.1097/MD.0000000000038705

**Published:** 2024-06-28

**Authors:** Can Cui, Zhu Fan

**Affiliations:** a Beijing Shijitan Hospital, Capital Medical University, Beijing, China; b Guang’anmen Hospital, China Academy of Chinese Medical Sciences, Beijing, China.

**Keywords:** Liang Xue Wu Hua Tang, molecular docking, network pharmacology, rosacea

## Abstract

Rosacea is a chronic and recurrent inflammatory skin disease affecting the center of the face that causes burning and itching sensations and changes in aesthetics. Liang Xue Wu Hua Tang (LXWHT) is a classic herbal formulation that is efficacious and has been widely used in the clinical treatment of rosacea; however, the pharmacological mechanisms remain unclear. The aim of the present study was to investigate the mechanism of action of LXWHT using network pharmacology and molecular docking. The Traditional Chinese Medicine System Pharmacology database was searched to identify the active ingredients and pharmacological targets of LXWHT, and the GeneCard, Disgenet, and Gene Expression Omnibus databases were applied to screen rosacea-related targets. Cytoscape software was used to visualize the protein–protein interaction network, and network topology analysis was used to identify core targets. Gene Ontology and Kyoto Encyclopedia of Genes and Genomes enrichment analyses were performed for the core targets. Molecular docking simulations and visualization were performed using Maestro and PyMOL, respectively. A total of 43 active compounds and 28 potential targets for LXWHT treatment of rosacea were selected for analysis. The Gene Ontology/Kyoto Encyclopedia of Genes and Genomes results indicated that LXWHT may exert therapeutic effects on rosacea by intervening in immune pathways including tumor necrosis factor pathway, interleukin-17 pathways, and Toll-like receptor signaling pathways. Chemokine ligand 2, interferon-γ, interleukin-1ß, peroxisome proliferator-activated receptor-γ, and matrix metallopeptidase 9 may be the core therapeutic target. Quercetin, stigmasterol, kaempferol, beta-sitosterol, luteolin, beta-carotene, baicalein, acetin, and isorhamnetin were predicted to be the key active ingredients. LXWHT may exert therapeutic effects in the treatment of rosacea by modulating immunity and angiogenesis, laying the foundation for further research.

## 1. Introduction

Rosacea is a chronic, recurrent, inflammatory dermatological condition that affects the center of the face and manifests as paroxysmal flushing, persistent erythema, papules and pustules, and capillary dilatation; these symptoms are often accompanied by burning and itching, as well as other discomforting sensations.^[1,2]^ The adverse impact on facial aesthetics often causes anxiety in patients, seriously affecting their social interactions and quality of life.^[3]^ There is no cure for rosacea, and most patients are dissatisfied with the results of treatments that are solely based on topical medications. In addition, some patients cannot tolerate the adverse effects of oral tetracyclines and retinoids. Therefore, pharmacotherapies that can safely and effectively treat rosacea are urgently needed.

Liang Xue Wu Hua Tang (LXWHT) is an effective formula for the clinical treatment of rosacea that consists of the following 5 traditional Chinese medicines: Carthamus tinctorius L. (Hong Hua [HH]), Celosia cristata L. (Ji Guang Hua [JGH]), Campsis grandiflora (Ling Xiao Hua [LXH]), Rosa rugosa Thunb. (Mei Gui Hua [MGH]), and Chrysanthemum indicum L. (Ye Ju Hua [YJH]). According to the traditional theory of Chinese medicine, the formation of rosacea is mainly due to dampness, heat, and blood stasis. The 5 herbs in LXWHT can play the roles of cooling the blood, activating the blood, dispersing wind, and removing toxins. Clinical studies indicate that the application of LXWHT alone or in combination with other agents can significantly reduce the clinical symptoms of rosacea, including facial itching, burning, flushing, and papules, without inducing significant adverse effects.^[4,5]^ However, the precise mechanisms of action and pharmacological effects of LXWHT remain largely unknown. Hence, there is an urgent need to identify the active components and explore the potential molecular targets of LXWHT in the treatment of rosacea.

The aim of the present study was to explore the mechanisms of action of LXWHT in the treatment of rosacea using network pharmacology to screen for the effective ingredients and core targets, as well as molecular docking experiments to verify the interactions between the core targets and the main effective ingredients.

## 2. Materials and methods

### 2.1. Network pharmacology

#### 2.1.1. Screening of active ingredients and targets of LXWHT

Each herbal medicine comprising LXWHT was entered into the Traditional Chinese Medicine System Pharmacology (TCMSP) database (https://old.tcmsp-e.com/tcmsp.php) one at a time, and the active ingredients of the herbal medicines were identified based on the following parameters: oral bioavailability ≥ 30%; and drug-like properties ≥ 0.18. The TCMSP database was subsequently searched to identify the targets of the active ingredients, which were then imported into the UniProt database (https://www.uniprot.org)^[6]^ to obtain the corresponding gene symbols.

#### 2.1.2. Screening of rosacea-related targets

The search term “Rosacea” was used to search the Gene Expression Omnibus database, and the GSE65914 gene microarray data were selected. The GEO2R online tool was selected to screen for differentially expressed genes (DEGs) between rosacea and normal tissues based on fold change and adjusted *P* values of |log_2_fold change| ≥1.2 and adjusted *P* < .05, respectively, as the screening criteria. Meanwhile, we searched the Disgenet database (https://www.disgenet.org/) and GeneCards (https://www.genecards.org) with “Rosacea” as the keyword, and screened the results with the following criteria: score > 0.1 in DisGeNET and score > 10 in GeneCards.^[7]^ We merged the screening results with DEGs as rosacea-related targets. We constructed Venn diagrams on the jvenn JavaScript library^[8]^ to identify overlapping target genes between LXWHT and Rosacea for further bioinformatics analyses.

#### 2.1.3. Construction of the “herb-component-target”(H-C-T) network

The intersection of LXWHT and rosacea target genes was assessed to identify potential targets of LXWHT in the treatment of the disease. More specifically, the herbs, active components, and potential targets of LXWHT were imported into the Cytoscape 3.8.2 software platform (The Cytoscape Consortium, San Francisco),^[9]^ and an H-C-T network diagram was constructed for visualization.

#### 2.1.4. Construction and analysis of the protein–protein interaction (PPI) networks

The intersection data of the LXWHT target genes and the rosacea target genes was input into the STRING database (https://string-db.org),^[10]^ with the biological species set to “Homo sapiens,” and the minimum required interaction score set to “medium confidence” (>0.4). The PPI network files were imported into Cytoscape software (version 3.8.2) for visualization. CytoHubba^[11]^ was used to calculate the degree centrality, closeness centrality, betweenness centrality, bottleneck, EcCentricity, and maximal clique centrality of the PPI network. The overlapping proteins between the top 10 protein lists obtained from each topological analysis were selected as the core targets, and a Venn diagram was constructed using the jvenn JavaScript library.

#### 2.1.5. Gene Ontology (GO) and Kyoto Encyclopedia of Genes and Genomes (KEGG) enrichment analyses

The intersecting genes were subjected to GO and KEGG signaling pathway enrichment analyses and visualized using the GO/KEGG analysis tool v0.2.0 from Hiplot website (https://hiplot.cn/)^[12]^ to generate a map of the potential signaling pathways through which LXWHT alleviates rosacea.

### 2.2. Molecular docking

#### 2.2.1. Preparation of key compounds and core protein files

The core proteins were identified from the construction and analysis of the protein interaction networks using the Protein Data Bank (http://www.rcsb.org/), and their 3D structures were saved as files in the Protein Data Bank format. The PubChem database (https://pubchem.ncbi.nlm.nih.gov/) was used to search for the key compounds, and their 2D structures were downloaded. The proteins and small molecules that were identified were subsequently imported into Schrödinger molecular docking software; protein receptors were prepared using the Protein Preparation Wizard module. All water molecules located >5 Å away from the active site were removed and incomplete amino acid residues were supplemented, het states were generated using Epik at pH = 7.0 ± 2.0. Hydrogen bonds were assigned via PROPKA at pH = 7.0. Energy was minimized using an OPLS3e force field until the relative mean SD between the minimized structure and the crystal structure exceeded 0.30 Å, and ultimately used as a receptor for molecular docking. Small molecule ligands were prepared using the LigPrep module (Schrödinger, Inc. New York City). The compounds were ionized and desalted at neutral pH 7 ± 2.0 with Epik, and energy was minimized using OPLS3e (Schrödinger, Inc. New York City).

#### 2.2.2. Preparation of molecular docking active pockets

The active pocket in the molecular docking experiments was defined by the ligands in the core proteins; in cases of proteins without ligands, the active binding sites were defined by the Binding Site Detection module in Schrödinger software, New York City. The binding grid is formed by the Glide module, which uses force-field information to produce frames indicating the possible locations of the ligand centers. The box was generated by directly selecting ligands from a known crystal structure or by simulating the active sites. The protein structure acts as a rigid receptor and each ligand molecule acts as a flexible ligand for semi-flexible docking. Standard Precision mode screening was performed for the docking of key components with defined active pockets, and the binding energy was calculated.

#### 2.2.3. Creation of a visual docking diagram

Small-molecule ligands and protein receptors were imported into PyMOL 2.4.0 software, (Schrödinger, Inc. New York City) to visualize the molecular docking results.

## 3. Results

### 3.1. Network pharmacology

#### 3.1.1. Screening results of the main active ingredients in LXWHT

**Figure 1.**
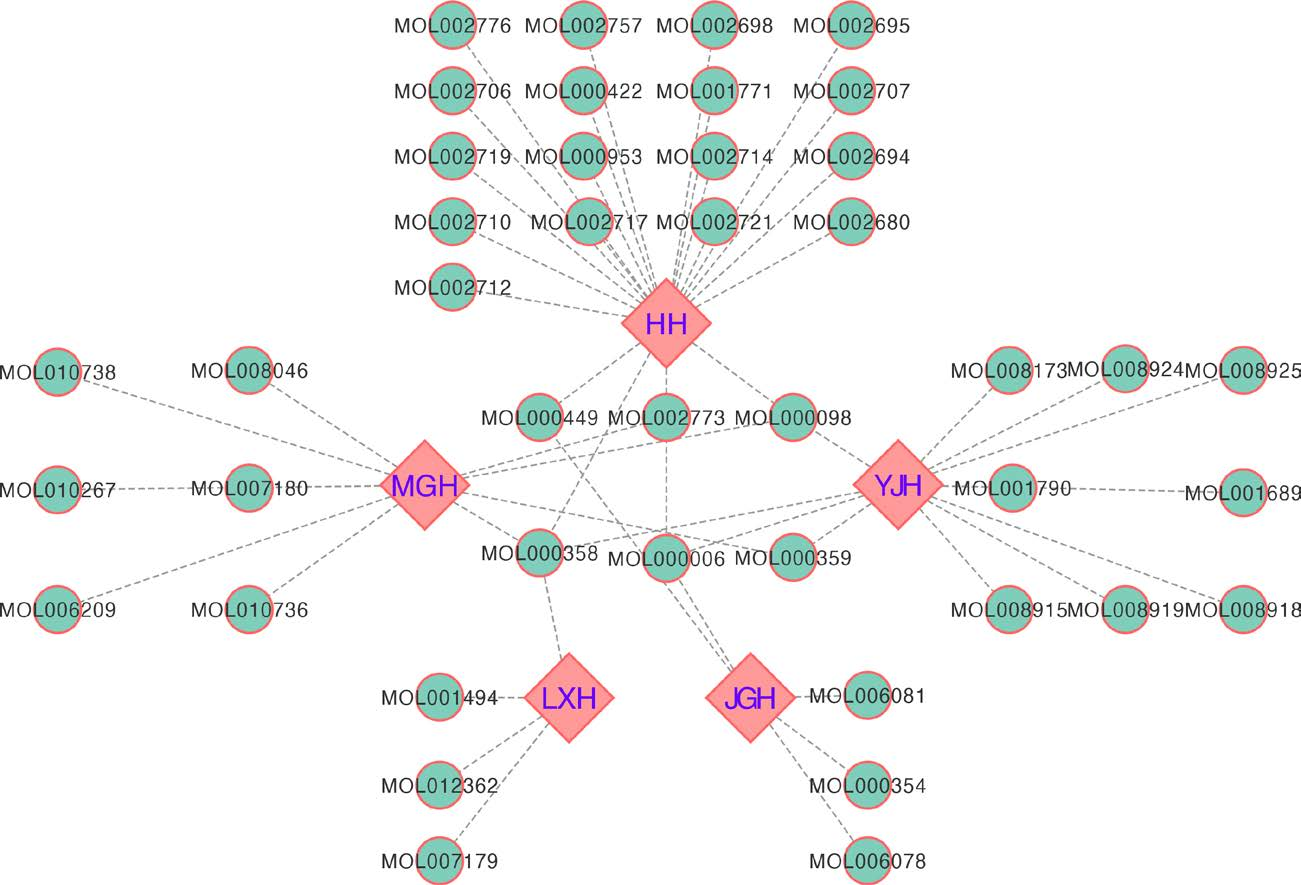
The “Herb-active Component” (H-C) network diagram of LXWHT. The diamonds represent the herbs comprising LXWHT, whereas the circles represent the active components. HH = Hong Hua, LXWHT = Liang Xue Wu Hua Tang, JGH = Ji Guang Hua, MGH = Mei Gui Hua, YJH = Ye Ju Hua.

The main active ingredients of the constituents of LXWHT were determined using the TCMSP database based on the oral bioavailability ≥ 30% and drug-like properties ≥ 0.18 criteria. A total of 43 main active ingredients were obtained from the search: 22 from HH, 12 from YJH, 5 from JGH, 4 from LXH, and 10 from MGH. These results were imported into Cytoscape to construct the “Herb-active Component” (H-C) network diagram of LXWHT, which is depicted in Figure 1.

The TCMSP database was subsequently used to identify the corresponding targets of the active ingredients, which were then imported into the UniProt website and converted into gene symbols. After eliminating duplicate results, 216, 191, 106, 37, and 178 targets were identified for HH, YJH, JGH, LXH, and MGH, respectively.

#### 3.1.2. Prediction of the therapeutic targets of LXWHT in the treatment of rosacea

**Figure 2.**
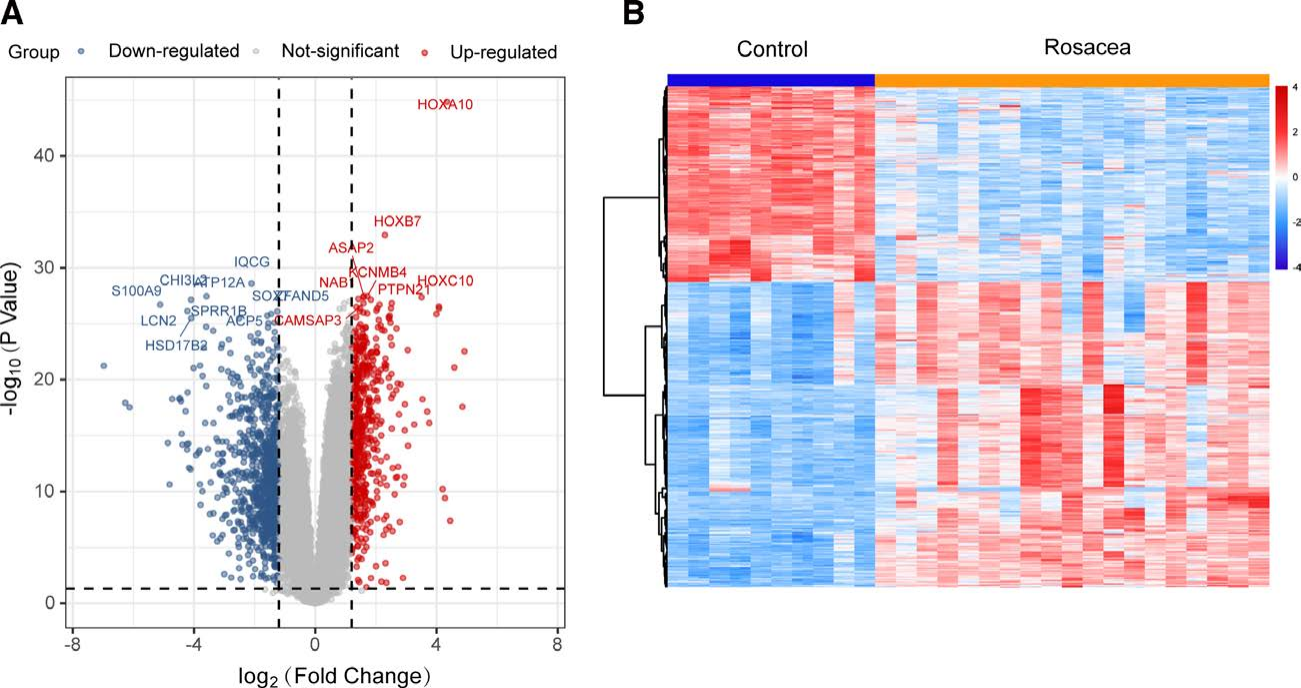
Demonstration of differentially expressed genes (DEGs) in GSE65914. (A) Volcano map and (B) Heatmap of DEGs.

**Figure 3.**
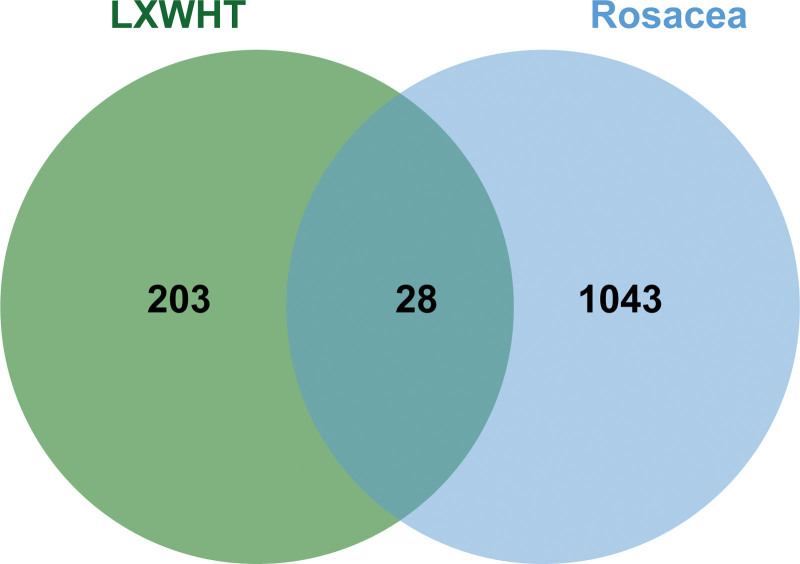
The Venn diagram identified the overlapping targets between Liang Xue Wu Hua Tang (LXWHT) and rosacea.

A total of 1070 DEGs were identified in rosacea tissues based on the GSE65914 dataset. We presented these DEGs with heatmaps and volcano plots (Fig. 2A and B). Further, we merged these DEGs with the screened genes from the GeneCard database and the Disgenet database to obtain a total of 1071 genes, that were considered to be rosacea-specific targets. Overlapping of these targets with the drug targets of LXWHT led to the identification of 28 intersecting targets, which were considered to be the therapeutic targets of LXWHT in the treatment of rosacea (Fig. 3).

#### 3.1.3. Construction and analysis of the “H-C-T” network of LXWHT in the treatment of rosacea

**Figure 4.**
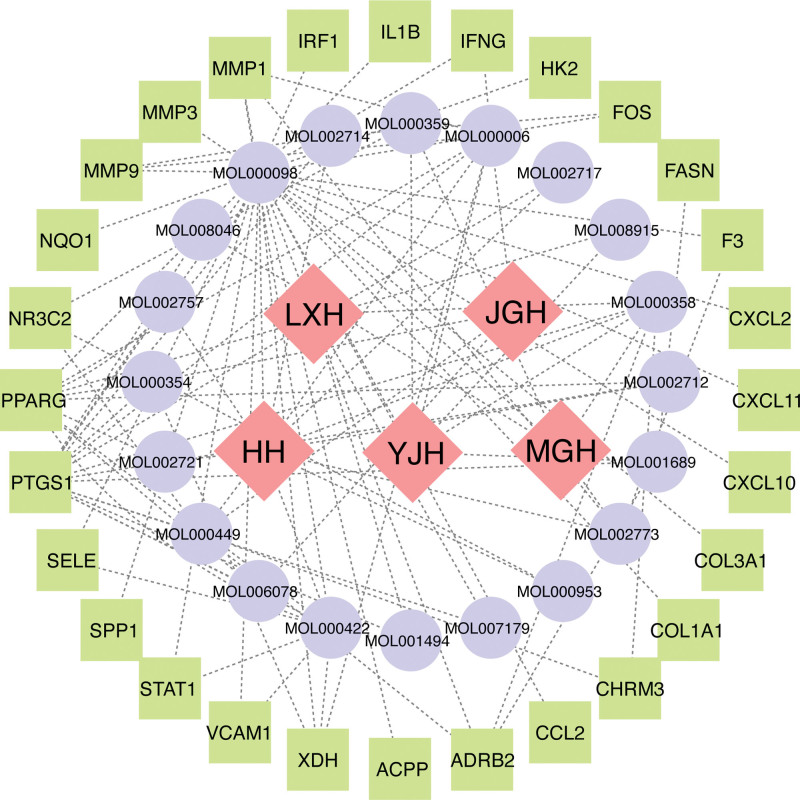
“Herb-Component-Target” (H-C-T) network of LXWHT in the treatment of rosacea. ACPP = acyl-carrier protein, ADRB2 = adrenoceptor beta 2, CCL2 = chemokine ligand 2, CHRM3 = cholinergic receptor muscarinic 3, COL1A1 = collagen, type I, alpha 1, COL3A1, collagen, type III, alpha 1, CXCL2 = C-X-C motif ligand 2, CXCL10 = C-X-C motif ligand 10, CXCL11 = C-X-C motif ligand 11, F3 = tissue factor III, FASN = fatty acid synthase, HH = Hong Hua, HK2 = hexokinase 2, IFNG = interferon-γ, IL1B = interleukin-1ß, IRF1 = interferon regulatory factor 1, LXWHT = Liang Xue Wu Hua Tang, JGH = Ji Guang Hua, MGH = Mei Gui Hua, MMP1 = matrix metallopeptidase 1, MMP3 = matrix metallopeptidase 3, MMP9 = matrix metallopeptidase 9, NQO1 = NAD(P)H quinone dehydrogenase 1, NR3C2 = nuclear receptor subfamily 3 group member 2, PPARG = peroxisome proliferator-activated receptor-γ, PTGS1 = prostaglandin-endoperoxide synthase 1, SELE = selectin E, SPP1 = secreted phosphoprotein 1, STAT1 = signal transducer and activator of transcription 1, VCAM1 = vascular cell adhesion molecule 1, XDH = xanthine dehydrogenase, YJH = Ye Ju Hua.

The herbs, active components, and 28 potential therapeutic targets of LXWHT were imported into STRING to construct the “H-C-T” network of LXWHT in the treatment of rosacea (Fig. 4).

Network topology analysis was further performed to calculate the degree value of each node in the network, with higher values indicating a closer connection with the surrounding nodes. Quercetin, kaempferol, luteolin, beta-sitosterol, stigmasterol, beta-carotene, baicalein, acacetin, and isorhamnetin exhibited degree values of 29, 9, 9, 7, 6, 4, 4, 4 and 4, respectively, all of which were higher than the median value; thus, they were considered to be the key active ingredients through which LXWHT alleviates rosacea. Detailed information for these key compounds is provided in Table 1.

**Table 1:** Information on key compounds of LXWHT.

Molecule	CID	Molecular weight	H-C-T network degree value
Quercetin	5280343	302.23 g/mol	29
Stigmasterol	5280794	412.7 g/mol	9
Kaempferol	5280863	286.24 g/mol	9
Beta-sitosterol	222284	414.7 g/mol	7
Luteolin	5280445	286.24 g/mol	6
Beta-carotene	5280489	536.9 g/mol	4
Acacetin	5280442	284.26 g/mol	4
Isorhamnetin	5281654	316.26 g/mol	4
Baicalein	5281605	270.24 g/mol	4

CID = compound identification number, H-C-T = herb-component-target, LXWHT = Liang Xue Wu Hua Tang.

#### 3.1.4. Construction and analysis of the PPI

**Figure 5.**
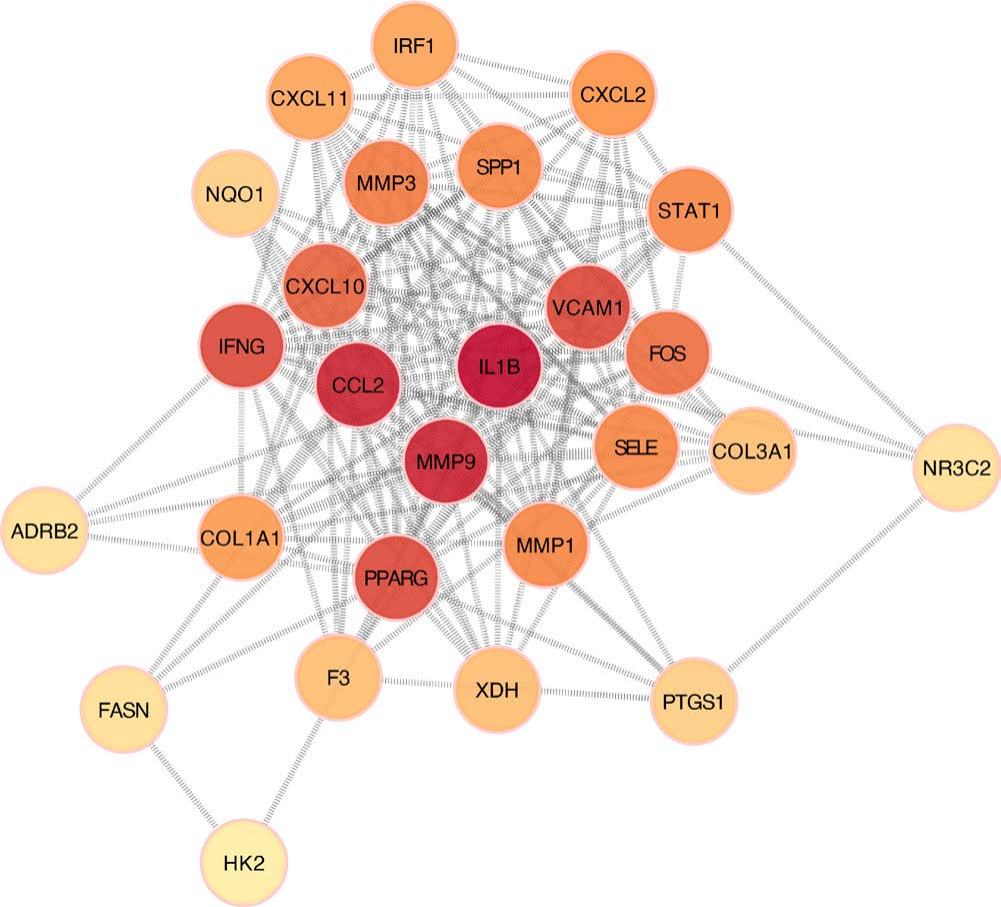
PPI network diagram of the intersecting targets. Nodes with a darker red color have a higher degree value and greater connectivity with surrounding nodes. ADRB2 = adrenoceptor beta 2, CCL2 = chemokine ligand 2, COL1A1 = collagen, type I, alpha 1, COL3A1 = collagen, type III, alpha 1, CXCL2 = C-X-C motif ligand 2, CXCL10 = C-X-C motif ligand 10, CXCL11 = C-X-C motif ligand 11, F3 = tissue factor III, FASN = fatty acid synthase, HK2 = hexokinase 2, IFNG = interferon-γ, IL1B = interleukin-1ß, IRF1 = interferon regulatory factor 1, MMP1 = matrix metallopeptidase 1, MMP3 = matrix metallopeptidase 3, MMP9 = matrix metallopeptidase 9, NQO1 = NAD(P)H quinone dehydrogenase 1, NR3C2 = nuclear receptor subfamily 3 group member 2, PPARG = peroxisome proliferator-activated receptor-γ, PPI = protein–protein interaction, PTGS1 = prostaglandin-endoperoxide synthase 1, SELE = selectin E, SPP1 = secreted phosphoprotein 1, STAT1 = signal transducer and activator of transcription 1, VCAM1 = vascular cell adhesion molecule 1, XDH = xanthine dehydrogenase.

The 28 intersecting targets were imported into the STRING database; after removing the isolated nodes, the PPI network was constructed and saved as a TSV-format data file, which was subsequently imported into Cytoscape 3.8.2 software for visualization (Fig. 5). The network comprised 27 nodes and 165 edges. Further network topology analysis was performed to calculate the Degree values of the nodes, with a darker red color indicating a higher degree value and greater connectivity with the surrounding nodes.

**Figure 6.**
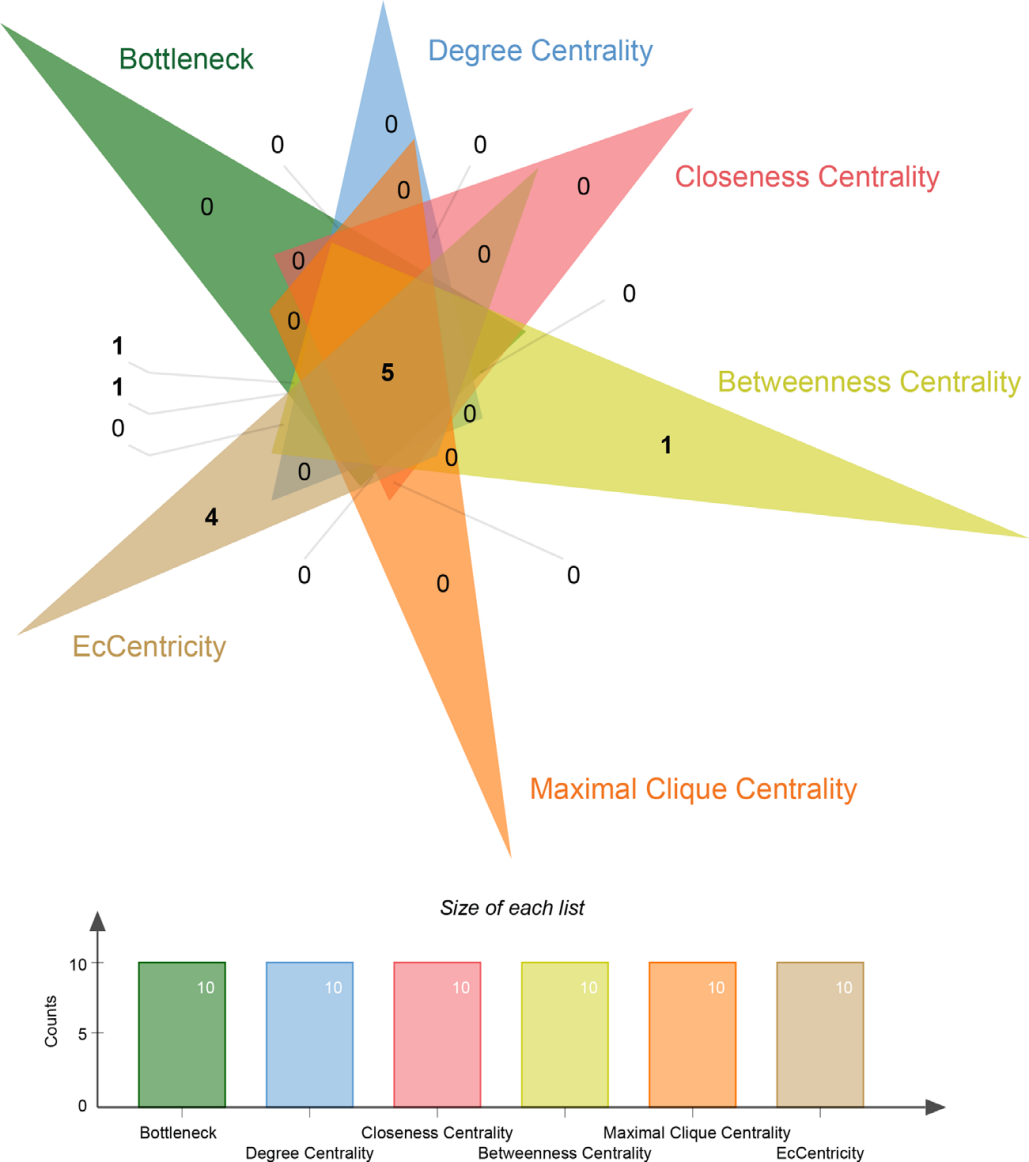
Screening of hub genes. Different colors represent different methods of topology calculation, here the top 10 genes under each algorithm are taken and they are subjected to a Veen diagram to take the intersection, the overlap is the intersecting genes under these 6 topology algorithms.

CytoHubba was used to topologically analyze the PPI network. The top 10 proteins calculated from each topological analysis were intersected, resulting in the identification of 5 intersecting proteins that were believed to be the key proteins involved in the pharmacological effects of LXWHT in the treatment of rosacea (Fig. 6), which included chemokine ligand 2 (*CCL2*), interferon-γ (*IFNG*), interleukin-1ß (*IL1B*), peroxisome proliferator-activated receptor-γ (*PPARG*), and matrix metallopeptidase 9 (*MMP9*).

#### 3.1.5. Enrichment analyses

**Figure 7.**
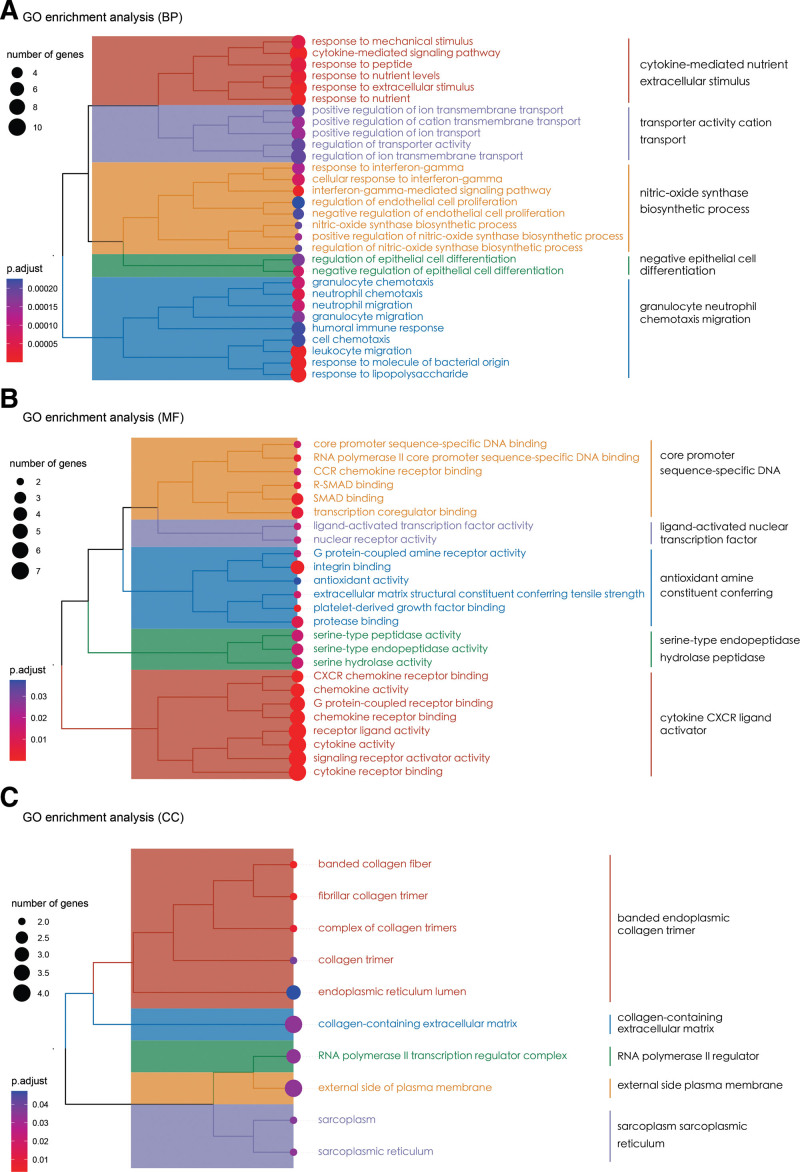
Cluster analysis of GO enrichment of core targets. (A) Biological process (BP) clustering diagram. (B) Molecular function (MF) clustering diagram. (C) Cellular composition (CC) clustering diagram. CCR = chemokine receptor, CXCR = C-X-C motif chemokine receptor, GO = Gene Ontology, R-SMAD = receptor-regulated Smad proteins.

GO enrichment analysis was performed on 28 intersecting targets to assess enrichment in categories related to molecular function (MF), biological processes (BP), and cellular composition (CC). According to the screening criteria of adjusted *P* < .05, 438 BP terms, 25 MF terms, and 10 CC terms were screened, clustered, and analyzed. In terms of the BP category, enrichment was predominantly observed in cytokine-mediated signaling pathways, transporter activity, nitric oxide synthase transport, activity transport, nitric oxide synthase biosynthetic processes, negative regulation of epithelial differentiation, and granulocyte and neutrophil chemotactic migration, among others. In terms of MF, enrichment was mainly observed in core promoter sequence-specific DNA binding, ligand-activated transcription factor activity, chemokine receptor binding, and cytokine receptor binding, among others. For the CC category, enrichment was observed for 10 terms, mainly including fibrillar collagen trimerization, banded collagen fibril, and a complex of collagen trimers. A cluster analysis was performed on the terms from each category, and the top 10 items were selected for visualization (Fig. 7).

**Figure 8.**
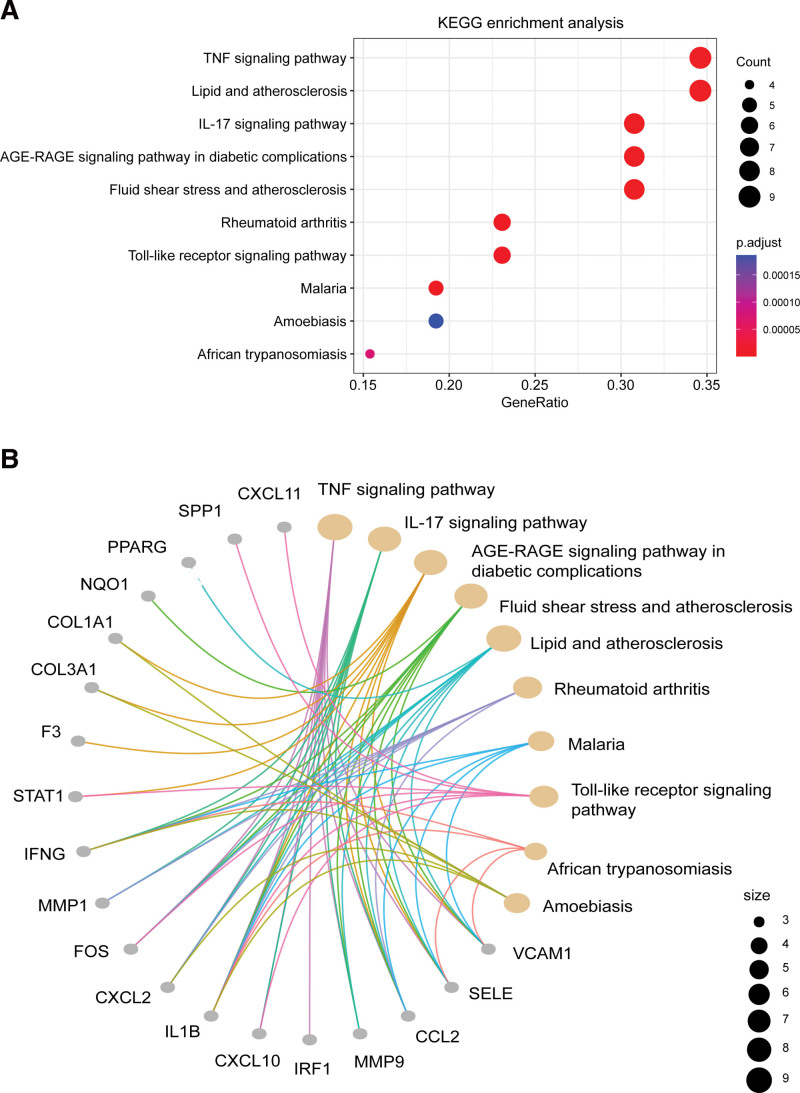
Bubble diagram (A) and pathway gene enrichment diagram (B) of the KEGG pathway enrichment analyses. ADRB2 = adrenoceptor beta 2, AGE-RAGE = advanced glycation end products-receptor for advanced glycation end products, CCL2 = chemokine ligand 2, COL1A1 = collagen, type I, alpha 1, COL3A1 = collagen, type III, alpha 1, CXCL2 = C-X-C motif ligand 2, CXCL10 = C-X-C motif ligand 10, CXCL11 = C-X-C motif ligand 11, F3 = tissue factor III, FASN = fatty acid synthase, HK2 = hexokinase 2, IFNG = interferon-γ, IL1B = interleukin-1ß, IL-17 = interleukin-17, IRF1 = interferon regulatory factor 1, KEGG = Kyoto Encyclopedia of Genes and Genomes, MMP1 = matrix metallopeptidase 1, MMP9 = matrix metallopeptidase 9, NQO1 = NAD(P)H quinone dehydrogenase 1, PPARG = peroxisome proliferator-activated receptor-γ, PTGS1 = prostaglandin-endoperoxide synthase 1, SELE = selectin E, SPP1 = secreted phosphoprotein 1, STAT1 = signal transducer and activator of transcription 1, TNF = tumor necrosis factor, VCAM1 = vascular cell adhesion molecule 1.

In terms of the intersecting targets from the KEGG pathway enrichment analysis, the top-ranked pathways included the tumor necrosis factor (TNF) signaling pathway, IL-17 signaling pathway, advanced glycation end products-receptor for advanced glycation end products signaling pathway in diabetic complications, fluid shear stress and atherosclerosis, lipids and atherosclerosis, rheumatoid arthritis, malaria, Toll-like receptor signaling pathway, African trypanosomiasis, amebiasis, and other pathways. The top 10 pathways are depicted in Figure 8A, and a protein crosstalk diagram for each pathway and its corresponding proteins is shown in Figure 8B.

### 3.2. Molecular docking of key components and core targets

The 5 core targets (*CCL2, IFNG, IL1B, PPARG*, and *MMP9*) were used as the receptors for molecular docking with the key active ingredients of LXWHT, and ligand-receptor docking scores were calculated; the smaller the docking core, the lower the binding energy and the more stable the conformation. Table 2 shows the 3 compounds with the lowest binding energies to the 4 targets of *CCL2, IFNG, PPARG*, and *MMP9* (the binding energies of the compounds to *IL1B* are not shown in Table 2 because they were all >−5.0).

**Table 2:** Docking scores of 4 of the core targets of LXWHT to the 3 compounds with the lowest binding energies.

Target	PDB ID	Compound name	Compound CID	Docking score	RMSD (Å)
IFNG	1eku	Kaempferol	5280863	−6.53	0.8774
		Acacetin	5280442	−6.41	1.1626
		Isorhamnetin	5281654	−6.35	0.67
PPARG	3GBK	Baicalein	5281605	−8.07	1.1962
		Acacetin	5280442	−7.78	0.8748
		Quercetin	5280343	−7.69	1.6696
MMP9	4H82	Luteolin	5280445	−7.46	1.5097
		Acacetin	5280442	−7.44	1.4184
		Kaempferol	5280863	−7.25	0.8016
CCL2	3IFD	Quercetin	5280343	−5.46	0.7935
		Kaempferol	5280863	−5.36	0.7942
		Isorhamnetin	5281654	−5.24	2.2949

CCL2 = chemokine ligand 2, CID = compound identification number, IFNG = interferon-γ, LXWHT = Liang Xue Wu Hua Tang, MMP9 = matrix metallopeptidase 9, PDB = protein data bank, PPARG = peroxisome proliferator-activated receptor-γ, RMSD = relative mean SD.

**Figure 9.**
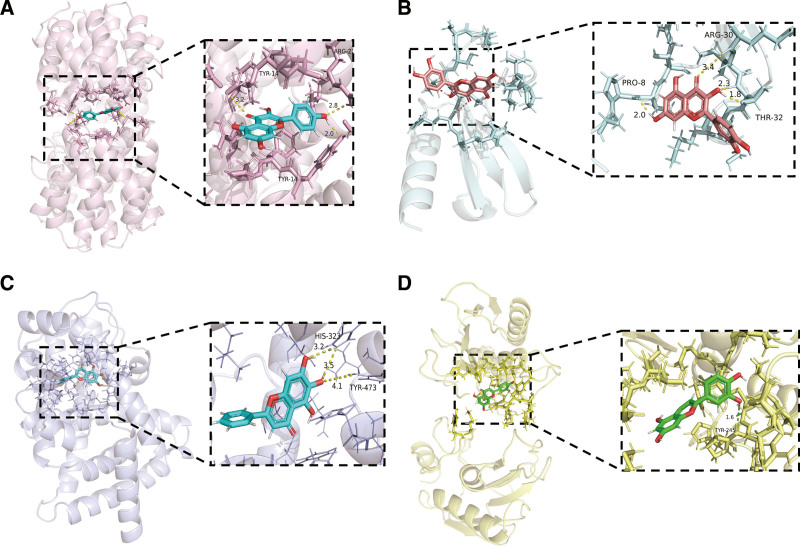
Molecular docking simulations between 4 of the key targets of LXWHT and the components with the lowest binding energy. (A) Docking between IFNG and kaempferol. (B) Docking between CCL2 and quercetin. (C) Docking between PPARG and baicalein. (D) Docking between MMP9 and luteolin. CCL2 = chemokine ligand 2, IFNG = interferon-γ, LXWHT = Liang Xue Wu Hua Tang, MMP9 = matrix metallopeptidase 9, PPARG = peroxisome proliferator-activated receptor-γ.

The docking scores of 4 of the core targets of LXWHT with the top 3 compounds were all below −5.0, indicating a high degree of binding and conformational stability. Among them, baicalein had the lowest binding energy of −8.07 with *PPARG*, and acacetin, quercetin, and kaempferol had low binding energies with several core targets, suggesting that acacetin, quercetin, kaempferol, and baicalein may be the core active compounds of LXWHT involved in the alleviation of rosacea. The lowest binding energy combinations for each core protein were selected for visualization using PyMOL 2.4.0 software (Schrödinger, Inc. New York City) (Fig. 9).

## 4. Discussion

The pathogenesis of rosacea is driven by a variety of factors, and, although the precise mechanisms are unknown, it is generally believed that a local immune imbalance, neurovascular dysfunction, and dermal microflora dysregulation occur mainly in individuals with a genetic susceptibility in response to certain external stimuli.^[13–15]^ Tetracyclines are efficacious in the clinical treatment of rosacea and can activate multiple anti-inflammatory and antioxidant pathways to reduce the associated clinical symptoms.^[16,17]^ However, rosacea is prone to recurrence, and tetracycline, as an antibiotic, can cause gastrointestinal reactions in some patients when administered for prolonged periods^[18]^ and drug resistance can develop. As an alternative, Traditional Chinese medicine exhibits great potential in the treatment of rosacea.

LXWHT is efficacious and has commonly been used in the clinical treatment of various facial erythematous diseases, including rosacea, facial dermatitis, and moderate-to-severe acne.^[19–21]^ In the case of rosacea in particular, LXWHT can significantly improve the symptoms of both paroxysmal and persistent erythema with a low incidence of adverse effects.^[4,5]^ Modern pharmacological studies indicate that Celosia cristata Flower extract can induce macrophages to produce nitric oxide synthase, cyclooxygenase-2, and cytokines, thereby modulating immune responses.^[22]^ The aqueous extract of *Chrysanthemum indicum L.* can achieve anti-inflammatory effects by regulating gut microbiota and short-chain fatty acids.^[23]^ Additionally, it can exert significant antibacterial effects against a wide range of bacteria, including *Staphylococcus aureus, Escherichia coli, Pseudomonas aeruginosa*, and *Candida albicans*.^[24]^ Hydroxysafflor yellow A, an active ingredient in *Carthamus tinctorius L*., can reduce inflammation by inhibiting *PI3K* and regulating *AKT*/*mTOR* and nuclear factor-κB (*NF-κB*) pathways in macrophages,^[25]^ and can inhibit angiogenesis by inhibiting *p38 MAPK* phosphorylation,^[26]^ although the specific mechanism of action remains unclear. Thus, in the present study, we explored the possible mechanisms of action of LXWHT in the treatment of the disease.

We identified 43 active compounds of LXWHT from the TCMSP database, and 28 putative therapeutic targets were selected based on the overlap between LXWHT-related targets and predicted rosacea targets. Based on the H-C-T network topology analysis, we identified several core compounds, including quercetin, stigmasterol, kaempferol, beta-sitosterol, luteolin, beta-carotene, baicalein, acetin, and isorhamnetin. Rosacea is a type of inflammatory disease, and many of these identified compounds are known to exhibit anti-inflammatory, antibacterial, and immunomodulatory properties. For example, quercetin, a flavonoid that acts on multiple targets to exert potent anti-inflammatory effects,^[27]^ can inhibit the levels of pro-inflammatory cytokines and other inflammatory mediators in skin lesions of patients with atopic dermatitis by upregulating *PPARα* and reducing the release of inflammatory factors from *IFN-γ*/*TNF-α*-treated human keratinocytes (Human adult low calcium high temperature cells).^[28–30]^ Stigmasterol, a natural steroid, also significantly reduces epidermal thickening and inflammatory cell infiltration in the skin of the ears of mice with irritant-induced dermatitis, while also reducing serum levels of *TNF-α*.^[31]^ Kaempferol has been shown to block 12-o-tetradecanoylphorbol-13-acetate-induced interleukin-1β expression in skin fibroblasts by inhibiting the phosphorylation of *NF-κB* and by modulating inhibitor of *NF-κB (IκBα*) through the inhibition of c-jun N-terminal kinase.^[32]^ There is also a plethora of clinical evidence suggesting that rosacea is associated with dysbiosis, and *Demodex folliculorum* mites, as well as *Helicobacter pylori, Staphylococcus epidermidis*, and *Chlamydia pneumoniae* are associated with the disease.^[14]^ Quercetin reduces H. pylori infection in the gastric mucosa and decreases both inflammatory responses and lipid peroxidation, in addition to disrupting biofilm formation by inhibiting the exopolysaccharide of Staphylococcus epidermidis.^[33,34]^ Luteolin, another key component identified in the present study, inhibits the growth of antibiotic-resistant strains of *H. pylori*^[35]^ Collectively, these studies suggest that the core compounds of LXWHT identified in the present study may be key substances involved in the treatment of rosacea.

After identifying 28 potential targets of LXWHT in the treatment of rosacea, a PPI network was constructed and topologically analyzed using CytoHubba. The top 10 targets ranked under each calculation method were selected, and 5 core targets were identified based on the intersection analysis, including *CCL2, IFNG, IL1B, PPARG*, and *MMP9*. Previous studies have shown that rosacea is mainly caused by persistent, aberrant innate immune responses that may be closely related to the functions of various immune cells.^[36]^ For example, macrophages may contribute to the pathogenesis of rosacea through various mechanisms that are mainly related to the release of inflammatory factors and the promotion of vasodilation.^[37,38]^ *CCL2* is an important cytokine that binds to *CCR2* and promotes monocyte/macrophage recruitment to lesion sites,^[39]^ while *IFNG* promotes macrophage activation, enhances antigen presentation, and coordinates innate immune system activation.^[40]^ A transcriptome sequencing study demonstrated that the *IFNγ*-mediated upregulation of the *STAT1*/*IRF1* signaling pathway in epidermal cells of rosacea lesions plays an important role in mediating epidermal–macrophage crosstalk and in regulating M1 polarization of macrophages.^[41]^ Activated macrophages express high levels of pro-inflammatory cytokines such as *IL-1β*, and mast cell activation and MMP release are another potential mechanism driving the pathogenesis of rosacea, as significant mast cell degranulation and increased expression of *MMP9* in rosacea lesions have been reported, the latter of which was significantly attenuated post-treatment.^[42]^ *PPAR-γ* is known to directly negatively regulate pro-inflammatory gene expression in a variety of immune cells in a ligand-dependent manner by antagonizing the activity of transcription factors such as *NF-κB* and members of the activator protein 1 family.^[29,43]^ The PPAR signaling pathway is downregulated in rosacea skin lesions, and the administration of an activator that targets *PPAR-γ* can reduce both erythema and vascular diastole.^[44]^ Therefore, LXWHT may exert anti-inflammatory as well as vascular remodeling and immunomodulatory effects through *CCL2, IFNG, IL1B, PPARG*, and *MMP9*, which are its core targets.

The results of the GO enrichment analysis of the core targets supported their involvement in anti-inflammatory, vascular remodeling, and immune regulatory pathways. In terms of the BP category, enrichment was mainly observed in the cytokine-mediated signaling pathway, transporter activity, nitric oxide synthase biosynthetic processes, negative regulation of epithelial cell differentiation, and granulocyte-neutrophil chemotactic migration, among others, suggesting that LXWHT may affect immune responses by interfering with the function and migration of neutrophils and lymphocytes. Nitric oxide biosynthetic processes are part of a classical pathway associated with vasodilator-related processes, which were also enriched, suggesting a potential mode of action of LXWHT. For the core targets in the KEGG analysis, enrichment was mainly observed in the TNF and *IL-17* signaling pathways, the advanced glycation end products-receptor for advanced glycation end products signaling pathway in diabetic complications, fluid shear stress, and atherosclerosis, Lipid and atherosclerosis, rheumatoid arthritis, malaria, and Toll-like receptor signaling pathways, provided further evidence of the potential anti-inflammatory and immunomodulatory effects of LXWHT. The Toll-like receptor signaling pathway is a classical innate immune pathway, and *TLR2*-mediated upregulation of kallikrein-5 activity in macrophages is important for the development of rosacea, as it leads to an increase in the cleavage of antimicrobial peptides and the formation of *LL-37*, a 37-amino acid peptide that promotes inflammation in the skin.^[45,46]^ In addition to natural immunity, acquired immunity is also involved in rosacea development. For example, the activation of T helper (Th) cells Th1 and Th17 in the area of skin lesions leads to the production of large amounts of IFN-γ and IL-17.^[47]^ These changes are associated with angiogenesis, inflammation, and the induction of *MMP9* expression, and *IL-17* can promote angiogenesis by inducing the upregulation of vascular endothelial growth factor through Janus kinase/*STAT* signaling.^[48,49]^ The present results confirm that the *IL-17* and TNF signaling pathways were significantly enriched. Collectively all these findings suggest that the compounds that comprise LXWHT target the TLR and IL-17 pathways to modulate the clinical symptoms of rosacea.

Ultimately, the topological analysis of the H-C-T network led to the identification of 5 hub genes (*CCL2, IFNG, IL1B, PPARG*, and *MMP9*). Further molecular docking simulations based on the predicted core compounds were performed to analyze the interactions between the protein receptors and their predicted active compounds. Except for those of *IL1B*, the docking scores between the remaining 4 core targets and their top 3 respective compounds were all below −5.0, suggesting good binding affinity. Among them, baicalein, acetin, quercetin, and kaempferol all exhibited low binding energies with several core targets, suggesting that these core components may alleviate the clinical inflammation of rosacea via binding to the aforementioned targets. However, the exploration of the pharmacological mechanism of action of LXWHT based on database searches and simulated docking alone is incomplete. Therefore, further experimental studies are required to verify these findings.

## 5. Conclusion

Collectively, the results of the present study demonstrate that LXWHT, a formulation used in traditional Chinese medicine, may have potential advantages in the treatment of rosacea owing to its anti-inflammatory, anti-proliferative, and immunomodulatory effects. However, further in-depth pharmacodynamic studies and molecular mechanism experiments are required to explore the full potential of LXWHT in the clinical treatment of rosacea.

## Author contributions

**Conceptualization:** Can Cui.

**Data curation:** Can Cui.

**Formal analysis:** Can Cui.

**Methodology:** Zhu Fan.

**Validation:** Can Cui, Zhu Fan.

**Writing – original draft:** Can Cui.

**Writing – review & editing:** Zhu Fan.
